# Atomistic Removal Mechanisms of SiC in Hydrogen Peroxide Solution

**DOI:** 10.3390/mi15060754

**Published:** 2024-06-03

**Authors:** Qin Man, Qiang Sun, Yang Wang, Jingxiang Xu

**Affiliations:** 1 College of Engineering Science and Technology, Shanghai Ocean University, Shanghai 201306, China; manq980412@foxmail.com (Q.M.); sunqiang@cicem.com.cn (Q.S.); 2 Research Institute of Frontier Science, Southwest Jiaotong University, Chengdu 610031, China; yang.wang@swjtu.edu.cn

**Keywords:** ReaxFF reactive force field, chemical mechanical polishing (CMP), SiC, polishing pressure

## Abstract

To elucidate the atomic mechanisms of the chemical mechanical polishing (CMP) of silicon carbide (SiC), molecular dynamics simulations based on a reactive force field were used to study the sliding process of silica (SiO_2_) abrasive particles on SiC substrates in an aqueous H_2_O_2_ solution. During the CMP process, the formation of Si-O-Si interfacial bridge bonds and the insertion of O atoms at the surface can lead to the breakage of Si-C bonds and even the complete removal of SiC atoms. Furthermore, the removal of C atoms is more difficult than the removal of Si atoms. It is found that the removal of Si atoms largely influences the removal of C atoms. The removal of Si atoms can destroy the lattice structure of the substrate surface, leading the neighboring C atoms to be bumped or even completely removed. Our research shows that the material removal during SiC CMP is a comprehensive result of different atomic-level removal mechanisms, where the formation of Si-O-Si interfacial bridge bonds is widespread throughout the SiC polishing process. The Si-O-Si interfacial bridge bonds are the main removal mechanisms for SiC atoms. This study provides a new idea for improving the SiC removal process and studying the mechanism during CMP.

## 1. Introduction

Silicon carbide (SiC) has excellent thermal conductivity, high electron drift rate, large lattice mismatch tolerance, and high breakdown field strength, and it is an excellent material for manufacturing high-power electronic devices and optoelectronic devices, like silicon carbon-based MOSFET and PiN [1]. In these electronic devices, it is critical to ensure that the SiC surface achieves an atomic-level, high-precision, damage-free surface, as the surface flatness and surface accuracy directly affect the quality of the SiC epitaxial layer, ultimately determining the performance of the device [2,3]. Therefore, the flattening of SiC surfaces has received extensive attention.

Chemical mechanical polishing (CMP) is widely recognized as a global flattening technique to achieve high-quality wafer surfaces. It has been applied to hard and brittle materials, such as silicon, copper, and glass [4,5,6,7]. Several researchers have deduced the material removal mechanism of SiC by examining the composition of the chemical components on the SiC surface before and after polishing. For example, Qi et al., using a scanning electron microscope and an energy dispersive spectrograph, measured the percentage of oxygen on the surface of SiC samples before and after dry CMP using five different solid-phase oxidants [8]. They deduced that the solid-phase oxidants facilitate the friction chemical reaction on the SiC surface under the effect of frictional heat, which generates an oxidation reaction film, promoting the oxidation of the silicon carbide surface. Consequently, it is inferred that under the impact of CMP, the solid-phase oxidizer can either oxidize the SiC surface with oxygen or engage in a chemical reaction with the SiC surface.

Furthermore, some researchers have experimentally investigated the effects of factors such as ultrasonic waves [9], abrasive concentration [10], abrasive particle size [11], temperature [12], pressure [13], and others in removing SiC materials. Shen et al. utilized XPS to infer the types of surface products and the potential chemical reactions occurring on SiC surfaces post-CMP based on spectral characteristics [14]. Although these macro-scale experiments effectively determine the process parameters affecting the CMP process, they do not clearly describe the mechanism of CMP for SiC due to the difficulty in observing the details of the SiC chemical reactions in real time. While XPS inspection can provide atomic details of the SiC CMP process, it cannot provide a dynamic view of atomic removal and thus fails to describe the mechanism of the process fully. However, understanding the removal mechanism is essential to improve the CMP process.

Molecular Dynamics (MD) simulation is a reliable tool for studying molecular motion from a microscopic perspective [15,16,17,18]. It has evolved into various theoretical systems to accommodate complex reactions in different environments. The commonly used MD is a simulation system based on Newtonian mechanics, which is suitable for analyzing intermolecular mechanical interactions. However, it fails to capture the state of intermolecular chemical reactions accurately. On the other hand, first-principle atomic dynamics can accurately describe the chemical reactions between atoms in computational systems [19,20,21]. However, the extensive computational cost associated with first-principal MD simulations, which involve many molecules, limits their application in CMP. In recent years, a new reactive force field (ReaxFF) MD simulation method was introduced to reflect the relationship between molecular movements based on changes at the bond level [22,23,24]. ReaxFF-MD simulations have been widely applied in various fields, such as physics, biology, and so on [25,26,27]. Ashish et al. reoptimized the ReaxFF parameters of the Si/O/C/H/N system to effectively address the stress analysis and temperature effect evaluation of SiC ceramic fibers [28]. These studies facilitate the development of the ReaxFF potential functions, expanding their application domains. Chen et al. employed the ReaxFF-MD method to investigate the oxidation behavior of different polar surfaces of 3C-SiC in aqueous solution and oxygen environments [29]. They accurately understood the system reaction activity and rate of SiC under various experimental conditions in the reaction and also clearly observed the formation mechanism of surface product SiO_2_. He et al. utilized the ReaxFF-MD simulation method to investigate the atomic oxidation, removal, and damage characteristics of nanoscale polished SiC substrates under the influence of chemical solution and abrasive vibrations [30]. They found that the vibration behavior of abrasives in vibration-assisted CMP enhanced the mechanical action, the atomic hybridization of SiC substrates, and the atomic activity, promoting the penetration of oxygen and hydrogen atoms. Meanwhile, this further promotes the adsorption of the chemical reaction between the solution and the substrate. According to Wu et al. [31], in their study of the mechanical properties of the oxide layer, the oxidation surface dramatically reduces the mechanical properties of the SiC surface. Yang et al. revealed that the H_2_O_2_ solution forms oxidation bonds on the 6H-SiC surface during the polishing process, thereby promoting the removal of Si and C atoms [32]. Thus, the oxidation of the SiC surface promotes the removal of atoms from the SiC surface. In this study, we will delve into how the oxidation of the SiC surface works during SiC CMP as well as the individual contributions of each removal mechanism to the total amount of substrate removal.

In this study, we utilized a previously developed transferable ReaxFF parameter set for carbon- and silicon-based solid materials. This parameter set was trained using a multi-objective simulated annealing algorithm and encompasses parameters for the elements H, C, O, and Si, covering a wide range of carbon/silicon-based solid materials. We employed ReaxFF-MD to investigate the atomic removal mechanism of SiC substrates by SiO_2_ abrasive polishing in CMP in hydrogen peroxide solution. During the CMP of SiC, the ionization and decomposition of H_2_O and H_2_O_2_ in the polishing solution will change the chemical properties of the SiC surface, resulting in a large number of H and O atoms on the SiC surface [33]. We explained the effect of the surface oxide layer on the removal of SiC from the surface material. Subsequently, we observed various SiC removal mechanisms and quantified their respective contributions to the overall removal process. The insights gained from this study enhance our understanding of the material removal process and mechanisms involved in SiC CMP, providing valuable guidance for optimizing CMP techniques in semiconductor fabrication.

## 2. Computational Details

In this study, we use a previously developed MD simulator (Laskyo) to perform the CMP process [34]. Moreover, ReaxFF is based on a bond-order reactive force field that can precisely represent bond formation and breakage. Thus, in this study, we employed the ReaxFF parameters developed in our previous study. These parameters effectively capture the interactions within the SiO_2_/water/SiC system [35]. All snapshots in this paper were generated by OVITO [36].

**Figure 1:**
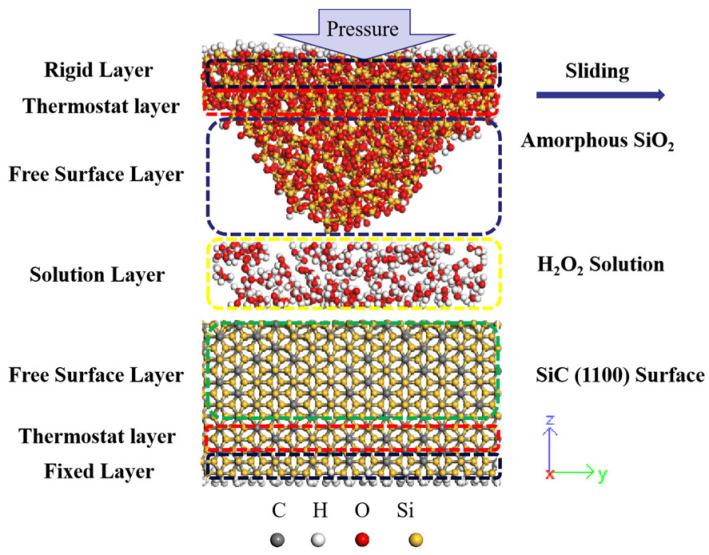
Schematic of the model for ReaxFF-MD simulations of SiC (1-100) CMP in aqueous H_2_O_2._

Figure 1 shows the simulation model for polishing the SiC slab with a single cluster of SiO_2_, which is widely employed as an abrasive grain [37,38]. In this study, we chose the 4H-SiC structure for constructing the SiC slab, because it has high electron mobility anisotropy, low mobility, and lower growth rate, making it more suitable for applying electronic components. Meanwhile, the SiC(1-100) surface (m-face) is employed as the polished surface because the (1-100) surface is the most stable crystalline surface compared to the SiC (0001) surface and the (000-1) surface. This stability plays an important role in manufacturing lateral SiC power devices [39]. The m-face of SiC crystals, comprising 1344 SiC atoms, is prepared using *NVP* simulation with the temperature set at 300 K. Temperature control is achieved using a *Berendsen* heat bath with a damping constant of 0.25 ps to minimize surface atomic energy [40]. We employed an aqueous solution containing 100 H_2_O molecules and 60 H_2_O_2_ molecules equilibrated by *NVT* simulation to accelerate the chemical reaction rate during the CMP process. To reduce the computational cost, we used a sine function to isolate an amorphous SiO_2_ structure using a sinusoidal function with a maximum value of 16 Å and retaining the 8 Å thickness of the flat plate above, which contains 513 Si atoms and 1026 O atoms. Next, the surface of the SiO_2_ particles was annealed using the *Nose-Hoover* thermostat to eliminate edge effects. This was achieved by subjecting the particle to *NVT* simulation, raising its temperature to 8000 K and then lowering it to 300 K to minimize potential energy through the rearrangement of surface atoms, a method similar to that used by Russo et al. [41]. Then, the SiC substrate, aqueous H_2_O_2_, and SiO_2_ were combined along the z-axis to generate the final simulation model, resulting in total system dimensions of 30.2 Å × 40.6 Å × 100 Å.

In Figure 1, the SiC surface model contains seven layers: (1) the fixed layer of the bottom SiC substrate atoms, which is constrained to be stationary over the entire simulation; (2) the thermostat layer of the SiC substrate, which is used to control the system temperature as constant; (3) the free SiC substrate layer; (4) aqueous H_2_O_2_ at the interface between the SiC substrate and SiO_2_ abrasive grain surfaces; (5) the free SiO_2_ abrasive grain layer, with atoms allowed to move dynamically in the simulations; (6) the thermostat layer of SiO_2_ abrasive grains; and (7) the rigid layer of the amorphous structure, which is laterally movable. The parameters set in this MD simulation are based on a metastable ReaxFF reaction model for carbon- and silicon-based solid materials. This model was previously developed and successfully applied in our previous aqueous H_2_O_2_ study.

All simulations are performed in the *NVT* ensemble with a time step of 0.25 fs. The *Nose-Hoover* temperature control method is employed to maintain the temperature at 300 K. The following five steps in the polishing process are conducted to simulate the sliding removal process: (1) To simulate the natural polishing environment more accurately, we initiate the α-SiO_2_/polishing solution/SiC system at a temperature of 300 k for 50 ps, allowing sufficient interaction between the system abrasive grains, the polishing solution, and the SiC substrate. (2) The normal load is uniformly applied to the top rigid layer of SiO_2_ along the z-axis. (3) The SiO_2_ moves vertically towards the surface of the SiC substrate, compressing the hydrogen peroxide solution at the interface until the target normal force matches the predetermined load to be applied to the SiO_2_. (4) The CMP simulation model is equilibrated for 50 ps. (5) SiO_2_ is slid laterally for 500 ps along the y-axis at a constant velocity of 100 m/s. In this study, a pressure of 2, 4, and 6 GPa is employed to investigate the effect of pressure on the CMP process. The specific simulation parameters are shown in Table 1.

## 3. Results and Discussion

### 3.1. Surface Oxidation of SiC in the Hydrogen Peroxide Solution

**Figure 2:**
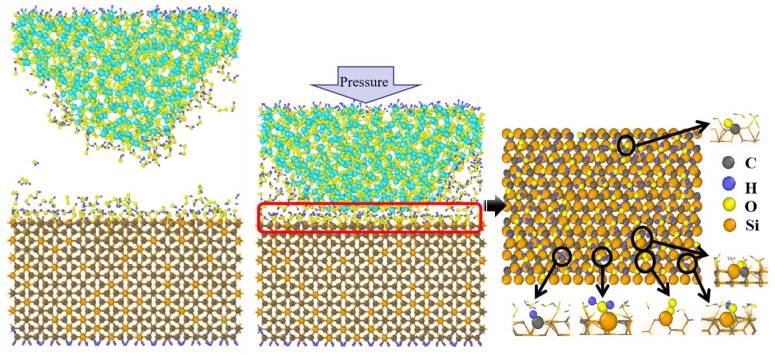
Atomic adsorption on the surface of SiC after sufficient reaction with the hydrogen peroxide solution. The red box shows the surface of the substrate after sufficient reaction, and the arrow points to a localized magnified view of the area of the red box.

Figure 2 illustrates the state of the polishing model at the end of relaxation and after loading, as well as the surface of the SiC after fully reacting with the aqueous H_2_O_2_. Figure 2 shows that the surface exhibits termination with Si-OH, Si-H_2_O, C-H, Si-O, and C-O-Si group structures. This is consistent with a previous study reported by Shen et al. [14]. H_2_O and H_2_O_2_ molecules undergo decomposition and ionization, generating numerous -H, -OH, -O, and other groups. These groups are adsorbed onto the surface of SiC, forming bonds such as Si-O-(O-) and C-O. This indicates that the SiC surface is oxidized after interaction with the aqueous H_2_O_2_, leading to surface softening, and the result is consistent with the literature [42].

**Figure 3:**
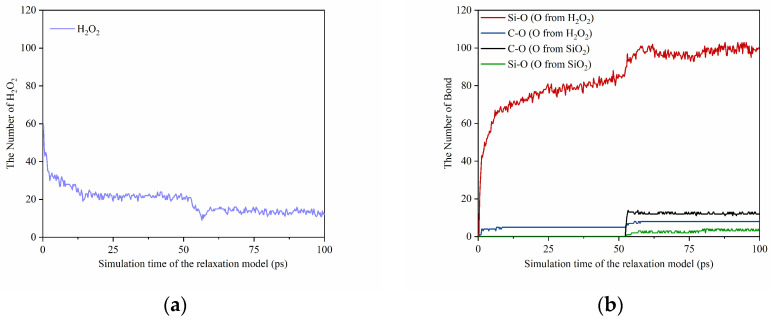
Variation in (**a**) the number of H_2_O_2_ molecules and (**b**) the number of oxidation bonds on the SiC surface during relaxation and loading.

**Figure 4:**
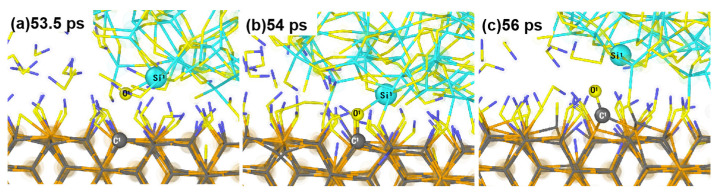
Bonding of O from SiO_2_ abrasive grains with SiC surface during loading.

To understand the effect of loading and hydrogen peroxide molecules on the oxidation of the SiC surface, we monitored the formation process of the surface structure and quantified the number of H_2_O_2_ molecules and main bonds (including Si-O and C-O) over the simulation time. As shown in Figure 3, during the first 50 ps, the substrate and abrasive grains remain stationary. The H_2_O_2_ solution at the interface (Figure 3a) reacts sufficiently with the abrasive grains and substrate to oxidize the substrate surface. Then, from 50–100 ps, we load the abrasive grains and performed relaxation calculations to construct the CMP model. Meanwhile, we find a further increase of the number of Si-O and C-O bonds (Figure 3b) on the substrate surface at this stage, and the SiC substrate forms a small number of Si-O and C-O bonds with SiO_2_ abrasive grains. Figure 2 shows that when the abrasive grain loading is complete, the bottom of the SiO_2_ surface undergoes plastic deformation due to extrusion. Figure 4 is a snapshot of the beginning of the loading phase for SiO_2_. We observe that during the loading process, O^1^ atoms from SiO_2_ are compressed to the SiC surface to form the O^1^-C^1^ bond with the C^1^ atom and attach to the substrate surface, facilitating the softening of the SiC surface. From Figure 3, it can be observed that the number of Si-O bonds formed on the surface of SiC after sufficient reaction with aqueous H_2_O_2_ is obviously higher than that of C-O bonds, indicating that the oxidation of Si atoms is the main form of SiC surface oxidation during the softening process of the SiC substrate.

### 3.2. Mechanisms for the Removal of SiC Material

**Figure 5:**
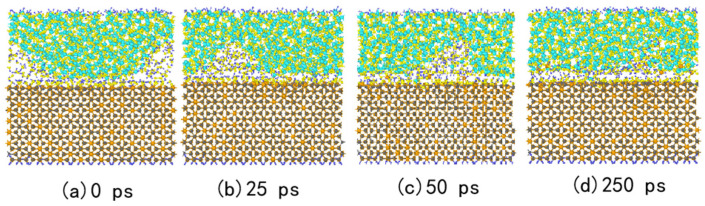
Snapshots for the shape variations of SiO_2_ during the polishing process.

To reveal the removal mechanism of the SiC surface, we tracked the breakage and formation process of their surface bonds and interfacial covalent bonds during the CMP process. It was found that the formation of Si-O-Si interfacial bridge bonds and the insertion of O atoms into the SiC surface can lead to the breakage of surface Si-C bonds, achieving atomic removal. Figure 5 presents some snapshots of the polishing process, from which we can observe deformation and even abrasion of the substrate as the abrasive grains continuously slide along the SiC surface. This is due to the fact that the hardness of SiO_2_ is much lower than that of SiC, and also because the SiO_2_ polishing process only removes the oxide layer of the substrate without causing subsurface damage (SSD) to the SiC surface. The detailed process is discussed in the following section using the example of a pressure of 6 GPa.

**Figure 6:**
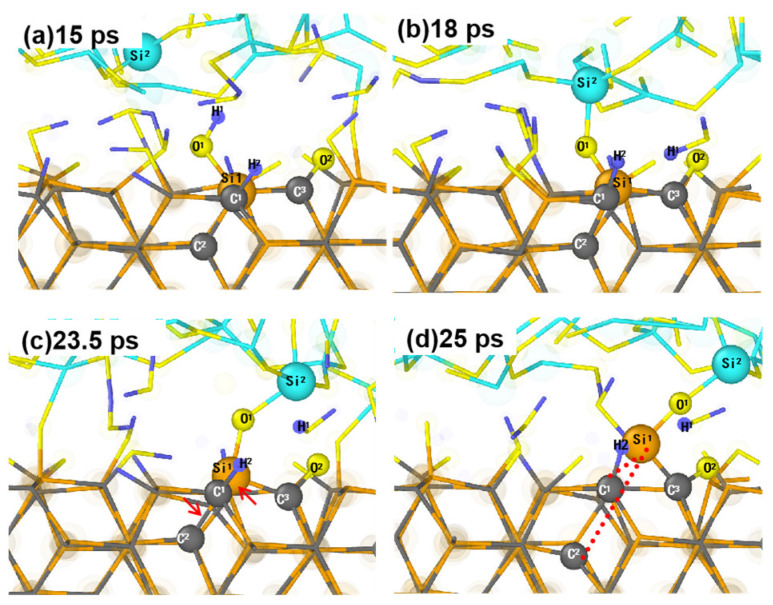
Snapshots for the breakage of the bonds on the SiC surface caused by the formation of Si-O-Si interfacial bridge bonds. The bond pointed to by the arrow is the one that is about to break, and the dotted line connects to the one that is already broken.

Figure 6 shows the process of removal of the Si atom from the SiC surface through an interfacial bridge bond. Before the sliding removal process, the Si^1^ atom belongs to the substrate and is bonded with three C atoms (C^1^, C^2^, and C^3^) and -O^1^H^1^ (Figure 6a). During the sliding process, the proton of H^1^ diffuses, and Si^2^ and O^1^ atoms form bridge bonds at the Si^1^-O^1^-Si^2^ interface, thus binding the abrasive grains to the substrate surface (Figure 6b). As the abrasive grains move, the Si^1^ atom is pulled up from the substrate surface because of the bridge bonding (Figure 6c). At 25 ps, the bonds of Si^1^-C^1^ and Si^1^-C^2^ are broken due to the tension generated by the continuous sliding of the abrasive grain, leading to the complete removal of the Si^1^ atom.

The formation of Si-O-Si interfacial bridge bonds is also observed during the loading process. However, there is no observed breakage of Si-C bonds in the region of the Si atoms forming the interfacial bridge bonds. Whereas, as the abrasive grains slide along the surface of the substrate when the Si-O-Si bonds are stretched to a certain extent, the breakage of the Si-C bonds is more likely to occur than for the Si-O bonds. This is because the previous experimental observations using angle-resolved X-ray photoelectron spectroscopy under water vapor plasma irradiation reveal that the bonding energy of the Si-C bond is lower than that of the Si-O [43], and thus, the activation energy for the breakage of the Si-C bond is much lower than the activation energy of the Si-O bond.

**Figure 7:**
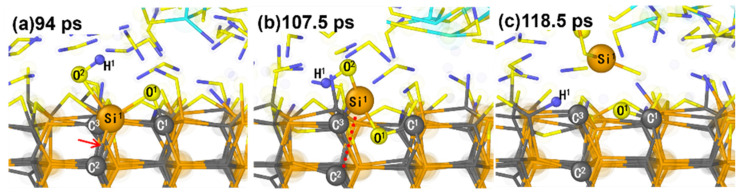
The snapshots of breakage of the bonds on the SiC surface arising from the insertion of oxygen atoms into the SiC surface. The bond pointed to by the arrow is the one that is about to break, and the dotted line connects to the one that is already broken.

Besides breaking the Si-C bonds due to the interfacial bridge bonds, which leads to the removal of Si atoms, the insertion of oxygen atoms also leads to the removal of Si atoms from the substrate. Figure 2 illustrates various oxygenated species on the SiC surface, such as Si-OH, Si-H_2_O, Si-O, and C-O-Si groups, resulting from interaction with an H_2_O_2_ solution. During the polishing process, O atoms from these species appear to insert into the surface, forming Si-O-C bonds (Figure 7). At 94 ps, the O^2^H^1^ is observed to adsorb on the Si^1^ atom of the SiC substrate. Meanwhile, the bond of Si^1^-O^1^-C^1^ is formed (Figure 7a). Then, the O^1^ atom inserts into the SiC surface under the pressure and sliding of the abrasive grains, and the lattice structure where the Si^1^ atom located is obviously deformed. At 107.5 ps, the O^1^ atom is entirely inserted into the substrate interior, while the Si^1^ atom is extruded. Additionally, this facilitates the easier removal of the Si^1^ atom protruding from the surface. As the sliding continues, the Si^1^-C^2^, Si^1^-O^1^, and Si^1^-C^3^ bonds break sequentially, completely separating the Si^1^ atom from the SiC surface (Figure 7c).

As depicted in Figure 7, inserting an O atom into the SiC surface leads to breaking the Si-C bond due to internal stresses. This causes local lattice deformation at the O atom’s location and protrusion of Si atoms from the surface. However, the Si-O-C bonds did not break during oxidation and pressure loading. This demonstrates that the removal of surface atoms due to the insertion of oxygen atoms demands the co-action of interfacial shear. This removal mechanism of SiC is similar to the removal mechanism of Cu surface materials. The previous studies for the removal mechanism of Cu atoms suggest that some copper atoms in the surface layer will oxidize and protrude from the surface after a certain reaction time and are then removed by the mechanical shear continuously generated by the sliding process of the abrasive grains [43,44].

**Figure 8:**
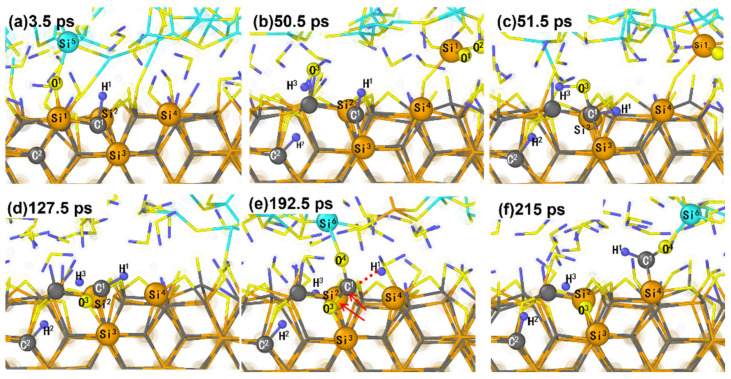
The snapshots of the removal of Si atoms destroying the stable structure of C atoms and inducing the removal of C atoms. The bond pointed to by the arrow is the one that is about to break, and the dotted line connects to the one that is already broken.

Next, we tracked the removal process of the C atoms. During the CMP process, it was observed that the chemical reactivity of C atoms is not as active as that of Si atoms. This makes it more difficult to remove C atoms than Si atoms during the processing of SiC surfaces. Figure 8 shows the removal process of the C atoms. Simulation results indicate that the removal of Si atoms in SiC changes the structural stability of the neighboring C atoms, reducing their strength and leading to subsequent removal (Figure 8).

Before the sliding removal process, the C^1^ atom belongs to the substrate and forms Si-C bonds with each of the three Si atoms (Si^1^, Si^2^, and Si^3^); the H^1^ atom is adsorbed on the surface of C^1^ to form a C^1^-H^1^ bond. At 3.5 ps, Si^5^ from the abrasive grain and O^1^ atom form a Si^5^-O^1^-Si^1^ bond under the combined effects of pressure and sliding of the abrasive grains. During the polishing process from 3.5 to 50.5 ps, the Si^1^ atom is pulled out of the SiC surface by the horizontal movement of the SiO_2_ abrasive grains due to the Si-O-Si interfacial bridge bond, making the surrounding structure of the original Si^1^ atom unstable. At 51.5 ps, the dissociation of the water molecule (H-O^3^-H^3^) generated O^3^H, which attached to the surface of the C^1^ atom to form a C^1^-O^3^H^3^ bond. During 51.5–127.5 ps, the O^3^ atom is inserted into the surface of the SiC substrate and combined with the Si^3^ atom to form the Si^3^-O^3^-C^1^ bond under the influence of the sliding motion of the abrasive grains. The originally stable tetrahedral lattice structure is apparently deformed after removal of the Si^1^ atom and the insertion of the O^3^ atom into the SiC surface. This also causes the C^1^ atom to protrude significantly from the SiC, making it much easier to remove. At 192.5 ps, the abrasive grains’ bond with C^1^ to form the Si^6^-O^4^-C^1^ interfacial bridge bonds, and with the continuous sliding of the abrasive grains to the right, the C^1^-O^3^ bond and the C^1^-Si^2^ bond are sequentially broken, resulting in the complete removal of the C^1^ atom from the SiC surface, as depicted in Figure 8f.

It can be seen from Figure 8 that the removal of Si atoms can lead to the removal of the C atoms next to them. Additionally, removing all other C atoms requires the removal of Si atoms first. Thus, it is concluded that C atoms are harder to remove than Si atoms. The Si atoms can be removed directly, whereas C atoms need to be structurally destabilized by the removal of Si atoms before they can be removed.

### 3.3. Effect of the Pressure on the SiC CMP Process

Pressure plays an important role in the material removal rate during the macro-scale CMP of various semiconductor materials. To further investigate the influence of pressure on the material removal, different pressures of 2, 4, and 6 GPa are set in our simulations. To quantify the degree of removal during the CMP process, we calculated the displacement of each atom in SiC during the sliding process,

(1) $$d=\sqrt{{\left(x_{0}-x_{1}\right)}^{2}+{\left(y_{0}-y_{1}\right)}^{2}+{\left(z_{0}-z_{1}\right)}^{2}\;}$$

In the CMP process, (x_0_, y_0_, z_0_) are the initial coordinates of the Si and C atoms, and (x_1_, y_1_, z_1_) are the position of the Si and C atoms. Previous studies have shown, through the analysis of the radial distribution function, that Si-C in SiC is no longer bonded when the distance is greater than 2.6 Å [15]. Thus, in this study, the amount of removal is defined by the number of SiCatoms with a displacement, d, greater than 2.6 Å during CMP.

**Figure 9:**
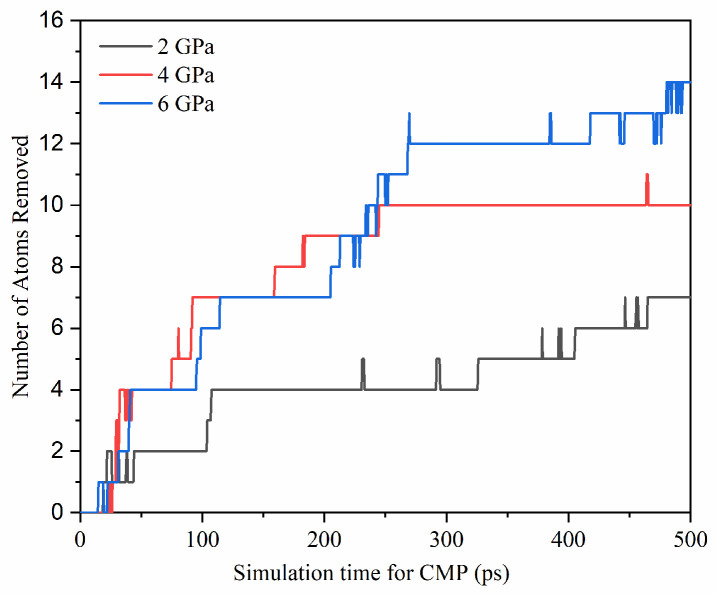
Variation of atom removal on the surface of SiC with polishing time under different pressures during the polishing process.

As depicted in Figure 9, the total removal of Si and C atoms during the CMP process of SiC gradually increases with polishing pressure. At 500 ps, the number of atoms removed at loads of 2, 4, and 6 GPa are 7, 10, and 14, respectively, with a linear growth trend. It is well known that the polished removal versus pressure and velocity, etc., can be expressed by Preston’s equation [45]:

(2) V=k×p×v×t

where k is the system parameter, p is the pressure, v is the velocity between the polishing disc and the wafer, and t is the polishing time. The removal is linearly related to the pressure when the velocity is constant. Our results are consistent with this trend of change. Moreover, this result aligns with the experimental findings of Zhou et al. [46] and Pan et al. [47] regarding the effect of polishing pressure on the removal of SiC materials, which demonstrated a positive correlation.

**Figure 10:**
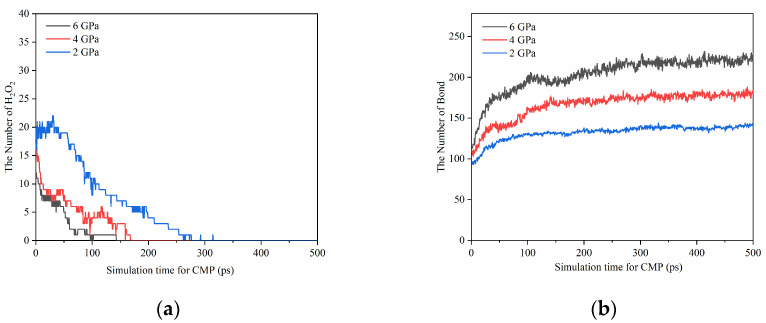
Variation of the amount of (**a**) H_2_O_2_ molecules and (**b**) oxidation bonds on the surface of SiC with polishing time under different pressures during the polishing process.

We investigated the effect of pressure on the chemical modification of the SiC surface during polishing by monitoring the variation of oxidation bonds and H_2_O_2_ molecules on the SiC surface at different pressures (Figure 10). The results indicate that with higher pressure, the total number of oxidation bonds on the SiC surface is higher (indicating a higher degree of oxidation on the SiC surface), which is more conducive to the removal of SiC atoms. In other words, pressure can promote the occurrence of chemical reactions on the polished surface.

**Figure 11:**
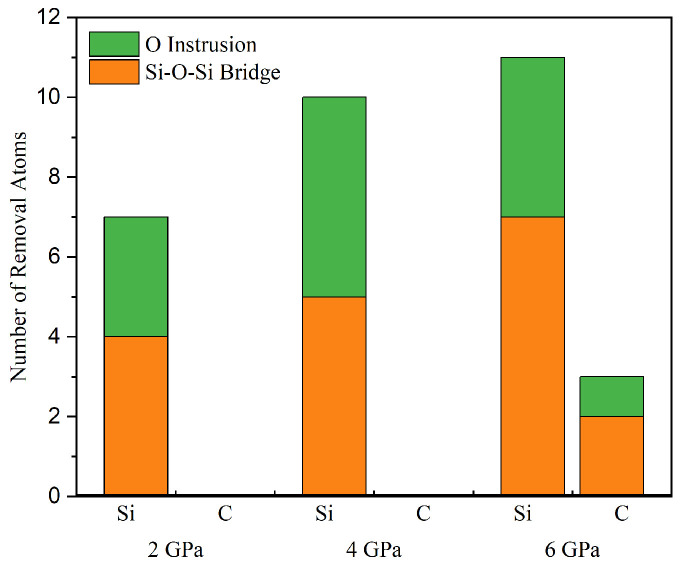
A number of silicon atoms were removed from the SiC surface due to different removal mechanisms after 500 ps of sliding at the pressures of 2, 4, and 6 GPa.

Furthermore, we distinguished the removal mechanisms leading to the removal of SiC atoms and quantified the number of atoms removed by different mechanisms to investigate the effect of pressure on the removal mechanisms (Figure 11). For the removal of Si atoms, it is observed that the removal of Si atoms is mainly caused by Si-O-Si interfacial bridge bonds and O insertion at each pressure. Under normal pressure, the contribution of different removal mechanisms to the removal of Si atoms from SiC surfaces is slightly higher for the Si-O-Si interfacial bridge bonds than for the type of removal mechanism of O insertion. Furthermore, the same trend exists under different loads. There is the same trend at different loads, which is more pronounced at higher loads. In addition, for the removal of the C atoms, it is found that when the pressure is less than 6 GPa, the C atoms are not completely removed during the simulation time, although Si atoms are removed. When the pressure level is 6 GPa, three C atoms are removed, and all of them are removed in such a way that the surrounding Si atoms are removed first, which leads to structural instability, thus making the C atoms easy to remove, as shown in Figure 8. These structures indicate that Si atoms can be removed directly during the CMP process, while carbon atoms are not easily removed. Meanwhile, increasing the pressure can promote the removal of C atoms. From Figure 10 and Figure 11, it is evident that the removal of SiC atoms results from both chemical reactions and mechanical loading, although chemical reactions play a critical and dominant role.

**Figure 12:**
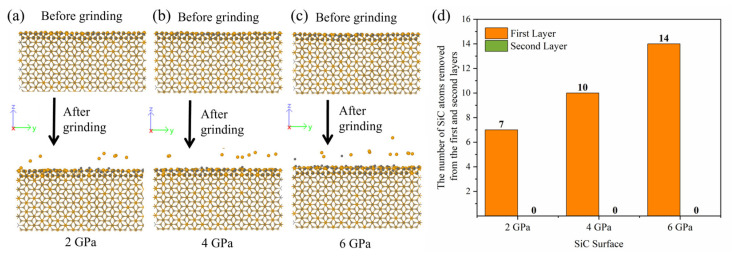
The snapshots of the atom removal from the first two layers of the silicon carbide surface at (**a**) 2 GPa, (**b**) 4 GPa, and (**c**) 6 GPa. The first two layers of atoms are shown normally. The rest of the atoms are transparent, showing only the chemical bonds. (**d**) The number of SiC atoms removed from the first and second layers.

In addition, the structure of the substrate surface after the CMP process is important, as it is often used to evaluate the effectiveness of the overall CMP process. We further analyzed the original positions of the removed atoms. Figure 12a–c shows that the removal behavior of SiC atoms only affects atoms in the first two layers of the substrate. As the pressure increases, an apparent local lattice distortion occurs on the polished surface, causing some atoms to protrude. To quantify the removal of atoms from each layer of the SiC surface, the initial origin of each removed atom is traced and statistically counted (Figure 12d). It can be observed that seven atoms are removed at 2 GPa pressure. In comparison, 10 and 14 SiC atoms are removed at 4 and 6 GPa pressure, respectively, belonging to the first layer. This result significantly impacts the realization of single-layer atom removal in ultra-precision machining.

## 4. Conclusions

The atomic mechanism of the CMP process on SiC surfaces polished by the abrasive grain of α-SiO_2_ is investigated by ReaxFF-MD simulations, and the following conclusions can be drawn:

(1) During the reaction, Si-OH, Si-H_2_O, C-H, Si-O, and C-O-Si groups are those mainly present on the substrate surface. There are two sources of -H_2_O, -O, and -OH in the above products: one from H_2_O molecules and one from H_2_O_2_ molecules. During the loading process, O atoms from the SiO_2_ abrasive grains combine with the SiC surface atoms under pressure, resulting in the formation of C-O and Si-O bonds that attach to the surface. This process further contributes to the softening of the substrate. Additionally, Si atoms on the substrate surface are more easily oxidized than C atoms throughout the process.

(2) Throughout the CMP process, the removal of SiC atoms is primarily governed by chemical reactions. Two fundamental mechanisms, namely the Si-O-Si interfacial bridge and O insertion, contribute to breaking surface bonds and completely removing SiC atoms. Additionally, because of the Si-O-Si interfacial bridge bonds and the sliding of SiO_2_ abrasive grains, mechanical shear plays a crucial supplementary role in facilitating the atom removal process. As a result, the removal of SiC atoms is a combined outcome of the synergistic effects of chemical reactions and mechanical loading.

(3) C atoms are more difficult to remove than Si atoms. The removal of Si atoms destroys the lattice structure of the substrate surface, resulting in a bump or even complete removal of the adjacent C atoms.

(4) Comparing the CMP process at different pressures, more SiC atoms are removed as the polishing pressure increases. We quantified the contribution of each removal mechanism to the removal of Si atoms from the SiC surface at different pressures and showed that the contribution of Si-O-Si bridge bonds at the interface between SiO_2_ and SiC is the largest, followed by O insertion. Moreover, this phenomenon is more pronounced at higher pressure. However, the mechanical action promoted the removal of atoms throughout the polishing process.

## Figures and Tables

**Table 1:** The specific simulation parameters.

Ensemble	NVT
Size of the model	30.2 Å × 40.6 Å × 100 Å
Sliding Time (ps)	500
Sliding speed (m/s)	100
Temperature (K)	300
Pressure (GPa)	2, 4, 6
Thermostat	Nose—Hoover
Timestep (fs)	0.25
Boundary Condition	X, Y are periodic boundaries, Z is stationary

## Data Availability

Data are contained within the article.

## References

[1] Zhang J., Zhang Y., Han J., He X., Yao W. Design and Fabrication of Large-Scale Lightweight SiC Space Mirror. Proceedings of the 2nd International Symposium on Advanced Optical Manufacturing and Testing Technologies.

[2] Dai S., Lei H., Fu J. (2020). Preparation of SiC/SiO2 Hard Core–Soft Shell Abrasive and Its CMP Behavior on Sapphire Substrate. J. Electron. Mater..

[3] Heydemann V.D., Everson W.J., Gamble R.D., Snyder D.W., Skowronski M. (2004). Chemi-Mechanical Polishing of on-Axis Semi-Insulating SiC Substrates. Mater. Sci. Forum.

[4] Lee H., Lee D., Jeong H. (2016). Mechanical Aspects of the Chemical Mechanical Polishing Process: A Review. Int. J. Precis. Eng. Manuf..

[5] Lee H., Park B., Jeong S., Joo S., Jeong H. (2009). The Effect of Mixed Abrasive Slurry on CMP of 6H-SiC Substrates. J. Ceram. Process. Res..

[6] Hara H., Sano Y., Mimura H., Arima K., Kubota A., Yagi K., Murata J., Yamauch K. (2006). Novel Abrasive-Free Planarization of 4H-SiC (0001) Using Catalyst. J. Electron. Mater..

[7] Aida H., Doi T., Takeda H., Katakura H., Kim S.W., Koyama K., Yamazaki T., Uneda M. (2017). Ultraprecision CMP for Sapphire, GaN, and SiC for Advanced Optoelectronics Materials. Curr. Appl. Phys..

[8] Qi W., Cao X., Xiao W., Wang Z., Su J. (2021). Study on the Mechanism of Solid-Phase Oxidant Action in Tribochemical Mechanical Polishing of SiC Single Crystal Substrate. Micromachines.

[9] Chen X., Liang Y., Cui Z., Meng F., Zhang C., Chen L., Yu T., Zhao J. (2022). Study on Material Removal Mechanism in Ultrasonic Chemical Assisted Polishing of Silicon Carbide. J. Manuf. Process.

[10] Chen X., Zhang C., Meng F., Yu T., Zhao J. (2023). Polishing Mechanism Analysis of Silicon Carbide Ceramics Combined Ultrasonic Vibration and Hydroxyl. Tribol. Int..

[11] Shi X., Pan G., Zhou Y., Gu Z., Gong H., Zou C. (2014). Characterization of Colloidal Silica Abrasives with Different Sizes and Their Chemical-Mechanical Polishing Performance on 4H-SiC (0001). Appl. Surf. Sci..

[12] Hoang T.T., Kutchma A.J., Becher A., Schweizer H.P. (2001). Effects of Process Parameter Variations on the Removal Rate in Chemical Mechanical Polishing of 4H-SiC. J. Electron. Mater..

[13] Zhou Y., Huang Y., Li J., Lv W., Zhu F. (2023). Polishing Process of 4H-SiC under Different Pressures in a Water Environment. Diam. Relat. Mater..

[14] Shen J., Chen H., Chen J., Lin L., Gu Y., Jiang Z., Li J., Sun T. (2023). Mechanistic Difference between Si-Face and C-Face Polishing of 4H–SiC Substrates in Aqueous and Non-Aqueous Slurries. Ceram. Int..

[15] Kang Q., Fang X., Wu C., Sun H., Tian B., Zhao L., Wang S., Jiang Z., Zhu N., Maeda R. (2021). Modification Mechanism of Collaborative Ions Implanted into 4H-SiC by Atomic Simulation and Experiment. Int. J. Mech. Sci..

[16] Li C., Piao Y., Meng B., Hu Y., Li L., Zhang F. (2022). Phase Transition and Plastic Deformation Mechanisms Induced by Self-Rotating Grinding of GaN Single Crystals. Int. J. Mach. Tools Manuf..

[17] Bai Y., Sui H., Liu X., He L., Li X., Thormann E. (2019). Effects of the N, O, and S Heteroatoms on the Adsorption and Desorption of Asphaltenes on Silica Surface: A Molecular Dynamics Simulation. Fuel.

[18] Zhang B., Kang J., Kang T. (2018). Effect of Water on Methane Adsorption on the Kaolinite (001) Surface Based on Molecular Simulations. Appl. Surf. Sci..

[19] Díaz Compañy A., Simonetti S. (2022). DFT Study of the Chemical Reaction and Physical Properties of Ibuprofen Sodium. Tetrahedron.

[20] He X., Chen M., Lv J., Xiao H., Wu H., Zhou R., Hu J., Zeng K., Yang G. (2023). A Theoretical Investigation on the Chemical Environment of Pyrazine-2,3-Dicarbonitrile and Phthalonitrile: Density Functional Theory (DFT) Calculation and Experimental Verification. J. Mol. Struct..

[21] Liu S.S., Zhang J., Xu Y., Yang Y., Xu P., Yu J.Y., Yang L.M., Liu W., Ni C.L., Zheng W.X. (2023). Structural, Vibrational, Optical Properties and DFT Calculations of a Zinc (II) Chloride Hybrid Based on Substituted Bipyridinium Cation. J. Mol. Struct..

[22] Van Duin A.C.T., Dasgupta S., Lorant F., Goddard W.A. (2001). ReaxFF: A Reactive Force Field for Hydrocarbons. J. Phys. Chem. A.

[23] Terzopoulou Z., Tsanaktsis V., Nerantzaki M., Achilias D.S., Vaimakis T., Papageorgiou G.Z., Bikiaris D.N. (2016). Thermal Degradation of Biobased Polyesters: Kinetics and Decomposition Mechanism of Polyesters from 2,5-Furandicarboxylic Acid and Long-Chain Aliphatic Diols. J. Anal. Appl. Pyrolysis.

[24] Jabraoui H., Gin S., Charpentier T., Pollet R., Delaye J.M. (2021). Leaching and Reactivity at the Sodium Aluminosilicate Glass–Water Interface: Insights from a ReaxFF Molecular Dynamics Study. J. Phys. Chem. C.

[25] Chen B., Diao Z.J., Lu H.Y. (2014). Using the ReaxFF Reactive Force Field for Molecular Dynamics Simulations of the Spontaneous Combustion of Lignite with the Hatcher Lignite Model. Fuel.

[26] Wen J., Ma T., Zhang W., van Duin A.C.T., Lu X. (2017). Atomistic Mechanisms of Si Chemical Mechanical Polishing in Aqueous H2O2: ReaxFF Reactive Molecular Dynamics Simulations. Comput. Mater. Sci..

[27] Yeon J., Van Duin A.C.T., Kim S.H. (2016). Effects of Water on Tribochemical Wear of Silicon Oxide Interface: Molecular Dynamics (MD) Study with Reactive Force Field (ReaxFF). Langmuir.

[28] Vashisth A., Khatri S., Hahn S.H., Zhang W., Van Duin A.C.T., Naraghi M. (2019). Mechanical Size Effects of Amorphous Polymer-Derived Ceramics at the Nanoscale: Experiments and ReaxFF Simulations. Nanoscale.

[29] Chen Z., Sun Z., Chen X., Wu Y., Niu X., Song Y. (2021). ReaxFF Reactive Molecular Dynamics Study on Oxidation Behavior of 3C-SiC in H2O and O2. Comput. Mater. Sci..

[30] He Y., Tang W., Gao P., Tang M., Fan L., Wang Y. (2023). Nano-Polishing Characteristics in Vibration-Assisted CMP of Single-Crystal Silicon Carbide via Molecular Dynamics Simulations. Mater. Sci. Semicond. Process.

[31] Wu Z., Zhang L. (2021). Mechanical properties, and deformation mechanisms of surface-modified 6H-silicon carbide. J. Mater. Sci. Technol..

[32] (2023). Shengyao Yang, Xuliang Li, Yitian Zhao, Md Al-amin, Lisbeth Grøndahl, Mingyuan Lu, Chi Fai Cheung, Han Huang, MD simulation of chemically enhanced polishing of 6H-SiC in aqueous H2O2. J. Manuf. Process..

[33] Su J., Du J., Ma L., Zhang Z., Kang R. (2012). Material Removal Rate of 6H-SiC Crystal Substrate CMP Using an Alumina (Al 2O 3) Abrasive. J. Semicond..

[34] Xu J., Higuchi Y., Ozawa N., Sato K., Hashida T., Kubo M. (2017). Parallel Large-Scale Molecular Dynamics Simulation Opens New Perspective to Clarify the Effect of Porous Structure on Sintering Process of Ni/YSZ Mul-ti-Particles, ACS Appl. Mater. Interfaces.

[35] Wang Y., Shi Y., Sun Q., Lu K., Kubo M., Xu J. (2020). Development of a Transferable ReaxFF Parameter Set for Carbon- And Silicon-Based Solid Systems. J. Phys. Chem. C.

[36] Alexander S. (2010). Visualization and Analysis of Atomistic Simulation Data with OVITO the Open Visualization Tool. Model. Simul. Mater. Sci. Eng..

[37] Liu W., Yuan S., Guo X. (2022). Atomic Understanding of the Densification Removal Mechanism during Chemical Mechanical Polishing of Fused Glass. Appl. Surf. Sci..

[38] Yu D. (2021). Single-Wafer Chemical Mechanical Planarization (CMP) on 150mm Silicon Carbide Substrate with Superior Surface Finish and Removal Efficiency. ECS Meet. Abstr..

[39] Chokawa K., Makino E., Hosokawa N., Onda S., Kangawa Y., Shiraishi K. (2019). Chemical vapor deposition condition dependence of reconstructed surfaces on 4H-SiC (0001), (000-1), and (1-100) surfaces. Jpn. J. Appl. Phys..

[40] Berendsen H.J.C., Postma J.P.M., Van Gunsteren W.F., Dinola A., Haak J.R. (1984). Molecular Dynamics with Coupling to an External Bath. J. Chem. Phys..

[41] Russo M.F., Li R., Mench M., Van Duin A.C.T. (2011). Molecular Dynamic Simulation of Aluminum Water Reactions Using the ReaxFF Reactive Force Field. Int. J. Hydrogen Energy.

[42] Deng H., Yamamura K. (2013). Atomic-Scale Flattening Mechanism of 4H-SiC (0001) in Plasma Assisted Polishing. CIRP Ann. Manuf. Technol..

[43] Wen J., Ma T., Zhang W., Van Duin A.C.T., Van Duin D.M., Hu Y., Lu X. (2019). Atomistic Insights into Cu Chemical Mechanical Polishing Mechanism in Aqueous Hydrogen Peroxide and Glycine: ReaxFF Reactive Molecular Dynamics Simulations. J. Phys. Chem. C.

[44] Guo X., Wang X., Jin Z., Kang R. (2018). Atomistic Mechanisms of Cu CMP in Aqueous H2O2: Molecular Dynamics Simulations Using ReaxFF Reactive Force Field. Comput. Mater. Sci..

[45] Preston F.W. (1927). The Theory and Design of Plate Glass Polishing Machines. J. Soc. Glass Technol..

[46] Zhou Y., Pan G., Shi X., Gong H., Luo G., Gu Z. (2014). Chemical mechanical planarization (CMP) of on-axis Si-face SiC wafer using catalyst nanoparticles in slurry. Surf. Coat. Technol..

[47] Ban X., Duan T., Tian Z., Li Y., Zhu J., Wang N., Han S., Qiu H., Li Z. (2023). Process optimization of 4H-SiCchemical mechanical polishing based on grey relational analysis. Semicond. Sci. Technol..

